# Transcatheter Closure of an Ascending Aortic Pseudoaneurysm Guided by Intracardiac Echocardiography

**DOI:** 10.7759/cureus.98755

**Published:** 2025-12-08

**Authors:** Manabu Sato, Hiromasa Hayama, Yae Matsuo, Yoshiyuki Yazaki, Kenji Makino, Go Hashimoto, Hidehiko Hara

**Affiliations:** 1 Division of Cardiovascular Medicine, Toho University Ohashi Medical Center, Tokyo, JPN; 2 Division of Cardiovascular Medicine, North Kanto Cardiovascular Hospital, Tokyo, JPN

**Keywords:** amplatzer vascular plug ii, aortic pseudoaneurysm, cardiac surgeries, case report, intracardiac echocardiography, percutaneous

## Abstract

A 60-year-old man with a history of multiple cardiac surgeries presented with exertional dyspnea. CT revealed a 20 × 10 mm ascending aortic pseudoaneurysm with a neck diameter of 7 mm, located near the suture line of a previous aortic root replacement, compressing a saphenous vein graft (SVG). Given the high risk of reoperation, percutaneous catheter closure with a 9/12 mm Amplatzer Vascular Plug II (AVP II) was performed alongside drug-eluting stent (4.0 × 18 mm) implantation to address SVG stenosis. Intraoperative intracardiac echocardiography (ICE) was essential for guiding AVP II deployment and ensuring accurate placement. This case highlights the effectiveness and safety of ICE-guided percutaneous interventions in managing complex aortic pathologies in high-risk patients. Successful closure of the pseudoaneurysm and resolution of the SVG compression demonstrated the feasibility of this minimally invasive approach in patients with complex surgical histories.

## Introduction

Ascending aortic pseudoaneurysm occurs in less than 0.5% of patients following cardiac surgical procedures [1]. Although rare, it is a potentially life-threatening condition. It can develop due to vascular wall injury from cardiac surgery and carries the risks of thrombosis, embolism, and rupture, which may be fatal, thus requiring therapeutic intervention. Surgical repair is the standard treatment [2]; however, reoperation carries significant risks, particularly for patients who have undergone multiple cardiac surgeries. In these cases, percutaneous catheter-based interventions are viable alternatives. Advances in percutaneous catheter techniques have reduced the morbidity and mortality associated with these procedures [3,4]. Previous case reports have demonstrated that the Amplatzer Vascular Plug II (AVP II) can be a safe and effective option for transcatheter closure of ascending aortic pseudoaneurysms, particularly in patients at high surgical risk [5-7].

Ascending aortic pseudoaneurysms are typically diagnosed by using multiple imaging modalities. A comprehensive evaluation of pseudoaneurysms and detailed anatomical features is essential for determining the appropriate catheter-based treatment strategy. When a catheter-based intervention is selected, intraoperative transesophageal echocardiography (TEE) is often performed in conjunction with fluoroscopic guidance for real-time monitoring [8]. However, visualization using TEE can be challenging due to prosthetic devices, such as mechanical valves, or anatomical alterations [9]. Intracardiac echocardiography (ICE) is a catheter-based ultrasound modality that allows real-time visualization from within the right atrium and helps identify cardiac and adjacent pericardiac structures.

This report demonstrates a successful case of percutaneous intraoperative ICE for an ascending aortic pseudoaneurysm, which enabled the avoidance of high-risk reoperation in a patient with a history of multiple cardiac surgeries.

## Case presentation

**Figure 1:**
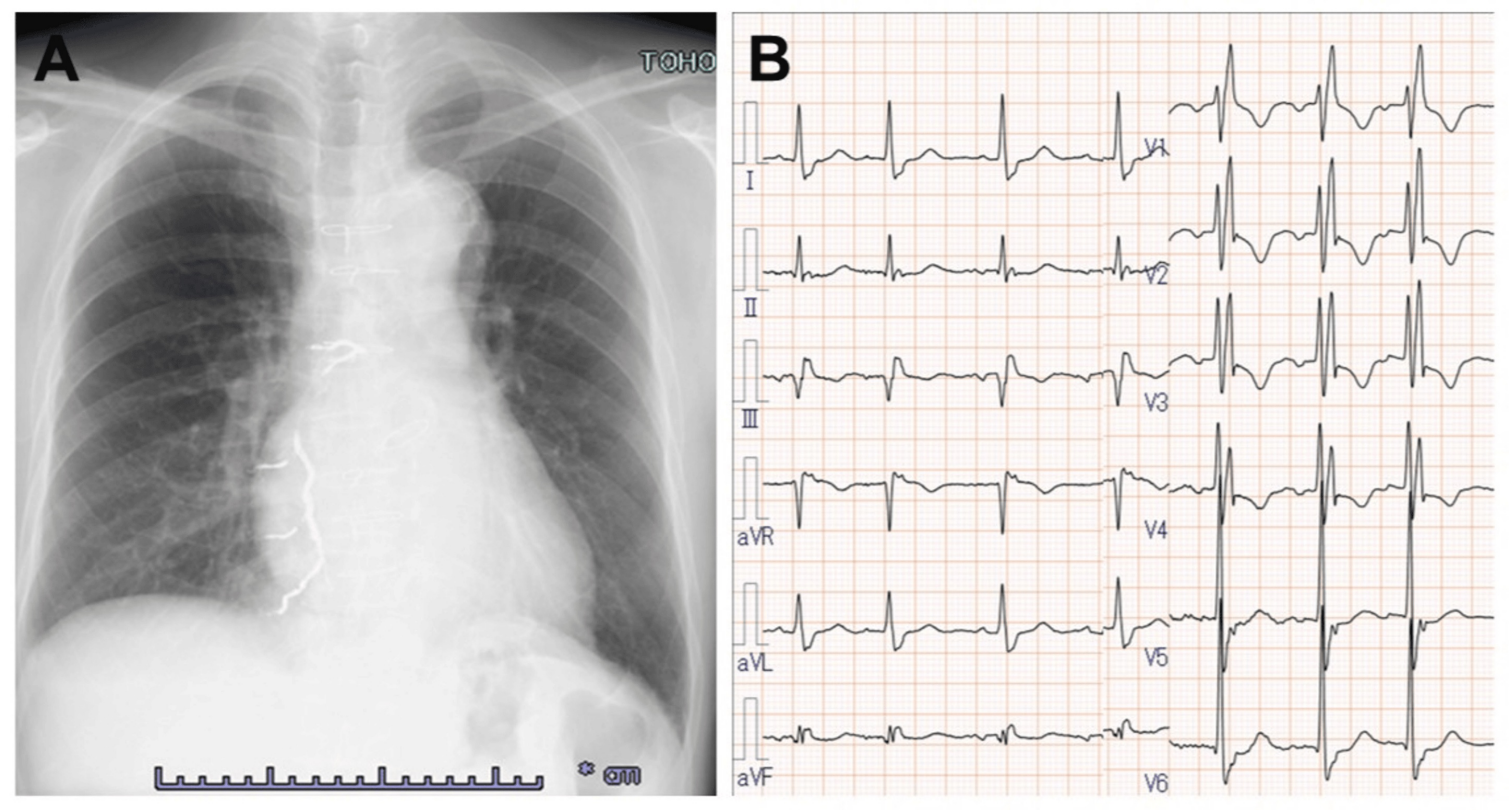
(A) Chest radiography showing a cardiothoracic ratio of 52% and evidence of previous bronchial artery embolization. (B) Electrocardiography indicating complete right bundle branch block and an abnormal Q wave in lead III.

**Figure 2:**
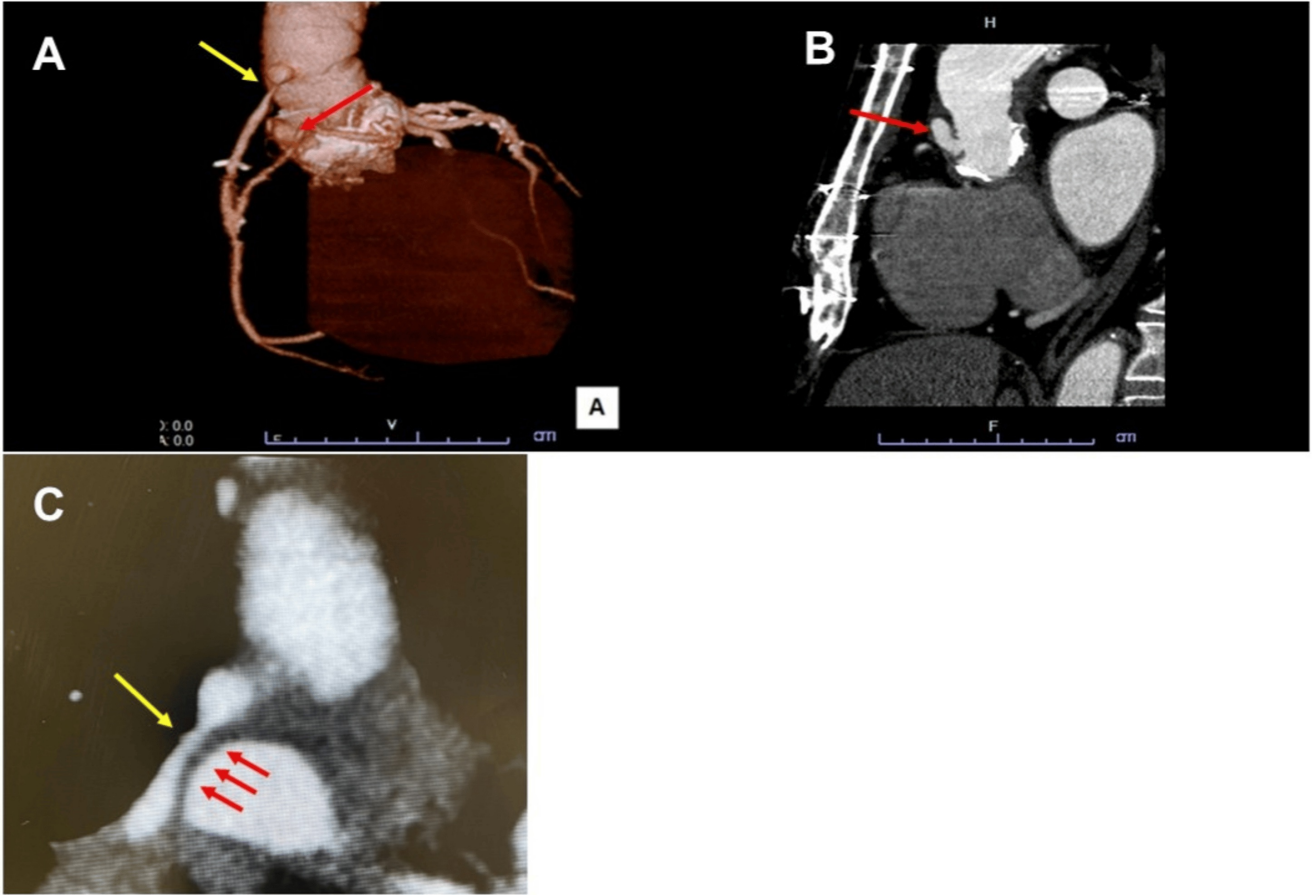
(A, B) CT scans showing an ascending aortic pseudoaneurysm (red arrow) with a neck diameter of 7 mm and associated SVG stenosis (yellow arrow). (C) CT scan showing the pseudoaneurysm (red arrow) compressing the SVG (yellow arrow); the pseudoaneurysm measured 20 × 10 mm. CT: computed tomography, SVG: saphenous vein graft

A 60-year-old man presented with exertional dyspnea. His medical history included multiple cardiac surgeries, including an initial aortic valve replacement 37 years previously, followed by a Bentall procedure 15 years previously. Six years prior, he underwent repeat aortic valve replacement due to bioprosthetic valve dysfunction, along with ascending aortic replacement and coronary artery bypass grafting. Postoperatively, mediastinitis developed, necessitating a re-sternotomy. Chest radiography revealed a cardiothoracic ratio of 52% and previous bronchial artery and right internal mammary artery embolization (Figure 1A). Electrocardiography revealed a complete right bundle branch block and an abnormal Q wave in lead III (Figure 1B). Contrast-enhanced CT revealed an ascending aortic pseudoaneurysm at the aortic root (Figure 2A), located at the suture line of the previous aortic root replacement, approximately 2 cm inferior to the SVG-aorta anastomosis, with a neck diameter of 7 mm (Figure 2B). The CT scan also revealed a 20 × 10 mm pseudoaneurysm that was compressing the SVG to the right coronary artery (Figure 2C), which was considered the cause of the patient’s exertional dyspnea.

Given his history of four open-heart surgeries and lung disease (aspergillosis) and a EuroSCORE II of 9.8%, reoperation was deemed high risk, and the patient was referred to our institution for percutaneous catheterization. As an antithrombotic therapy associated with the procedure, clopidogrel 75 mg daily was added to the ongoing warfarin 2 mg daily regimen.

Procedure details

**Figure 3:**
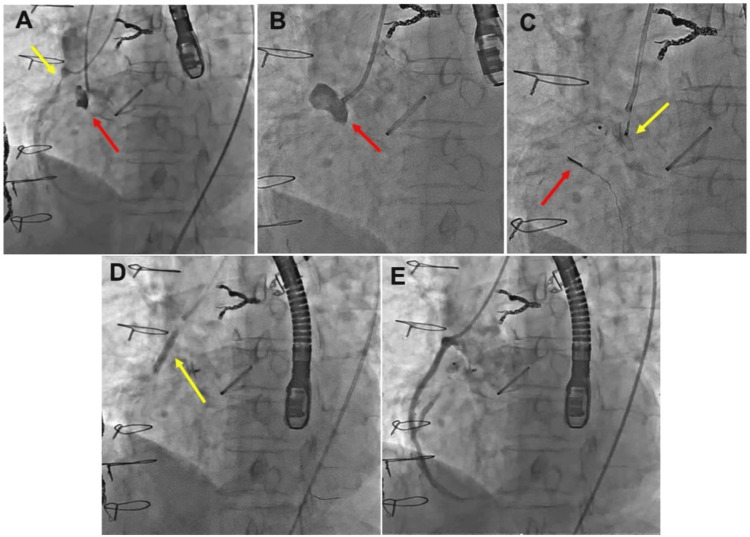
(A) Simultaneous angiography to delineate the anatomy, showing 90% stenosis of the SVG (yellow arrow), aortic pseudoaneurysm (red arrow), and the anatomy of the mechanical valve. (B) Angiography to evaluate the morphology of the aortic pseudoaneurysm (red arrow). (C) Placement of the AVP II 9/12 mm (yellow arrow) under ICE guidance (red arrow). (D, E) Stent placement and patency in the SVG stenosis. SVG: saphenous vein graft, AVP II: Amplatzer Vascular Plug II, ICE: intraoperative intracardiac echocardiography

CT revealed a 6-mm rim at the pseudoaneurysm's entry point near the mechanical valve, with a neck diameter of 7 mm. Based on the rim morphology and neck diameter, we determined that a plug device was feasible and selected an AVP II with a 9/12 mm (disk diameter 12 mm) configuration. Coils were also prepared as backups in the case of a device mismatch. We used a 6-Fr Judkins Right 4.0 guiding catheter with side holes and a 4-Fr pigtail catheter to access the pseudoaneurysm. Simultaneous angiography was performed to assess the anatomical relationship between the pseudoaneurysm and SVG (Figure 3A). Angiography of the pseudoaneurysm confirmed its morphology (Figure 3B). ICE and fluoroscopic imaging confirmed that the device was securely positioned and did not interfere with the mechanical valve, and the AVP II was successfully deployed into the pseudoaneurysm (Figure 3C). In addition, a drug-eluting stent (Ultimaster Nagomi 4.0 × 18 mm) was implanted in the SVG to treat the 90% stenosis (Figure 3D). IVUS was used to optimize stent sizing and deployment. Post-stent angiography revealed satisfactory expansion of the SVG (Figure 3D).

**Figure 4:**
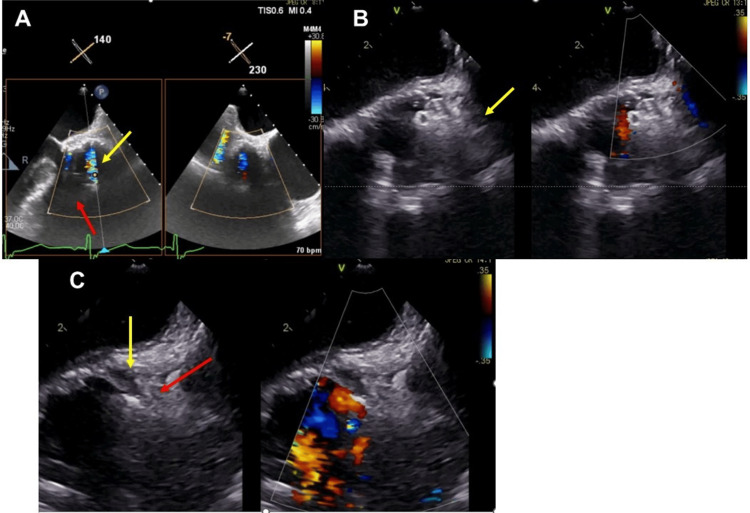
(A) TEE image showing shunt flow into the pseudoaneurysm (yellow arrow), but evaluation is limited by artifacts from the mechanical valve (red arrow). (B) ICE image demonstrating clear visualization of the pseudoaneurysm (yellow arrow) and surrounding anatomical structures. (C) Placement of the AVP II under ICE guidance, with confirmation of device visualization (red arrow) and adequate rim capture (yellow arrow). TEE: transesophageal echocardiography, ICE: intraoperative intracardiac echocardiography, AVP II: Amplatzer Vascular Plug II

**Figure 5:**
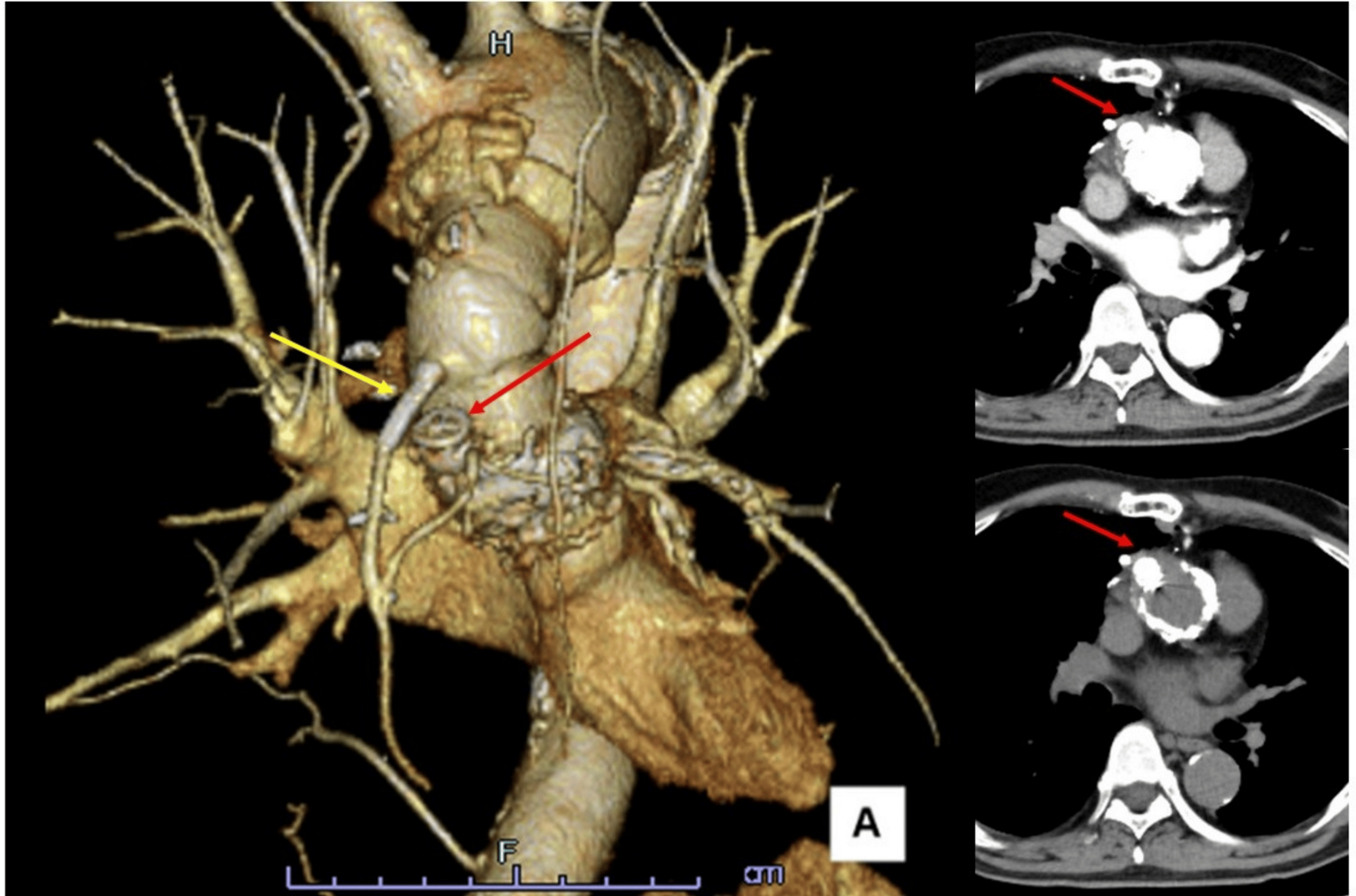
Postoperative CT (3D and contrast-enhanced CT) images demonstrating optimal placement of the stent (yellow arrow) and AVP Ⅱ (red arrow). The high-density structure observed in the contrast-enhanced CT images corresponds to the AVP II. CT: computed tomography, 3D: three-dimensional, AVP Ⅱ: Amplatzer Vascular Plug II

Initially, TEE was performed under intraoperative imaging guidance. However, while color Doppler on TEE detected a shunt flow into the pseudoaneurysm, the surrounding structures were unclear due to artifacts from the mechanical valve (Figure 4A). Because precise device placement is challenging, ICE was performed via venous access, and the device was positioned in the right atrium. ICE confirmed that the pseudoaneurysm on the right coronary cusp side was visualized in the short-axis view of the aorta (Figure 4B). Intraoperative ICE imaging confirmed correct placement and stability of the device. Furthermore, ICE allowed us to verify rim capture during the AVP II deployment, ensuring safe placement (Figure 4C). Postoperative CT confirmed that the AVP II was securely placed with no contrast leakage into the pseudoaneurysm, and the stent in the SVG remained patent (Figure 5).

## Discussion

The management of ascending aortic pseudoaneurysms presents a significant challenge in patients who have undergone multiple prior cardiac surgeries. Traditional surgical repair is associated with a substantial risk, particularly in those who have experienced several cardiac procedures. The complexity of these cases increases the likelihood of complications such as bleeding, infection, and organ dysfunction. In high-risk patients, percutaneous catheter-based treatments offer a minimally invasive alternative with shorter recovery times and lower complication rates [3,4].

The selection of an appropriate closure device is determined by the size, location, and anatomical relationship of the pseudoaneurysm and its surrounding structures [10-12]. The available options for catheter-based treatments include stent grafts, coils, and occlusion devices. In this case, we selected an AVP II because it was considered more reliable and complete for occluding a medium-to-large pseudoaneurysm with a relatively narrow neck compared to coils. Additionally, a stent graft was considered less suitable due to its close proximity to the SVG, making a plug device the safer option in this anatomical context. The AVP II cap technique facilitates efficient occlusion of the pseudoaneurysm while sealing its entry point, reducing the risk of residual flow or rupture [13]. Additionally, a drug-eluting stent was implanted to address significant stenosis in the SVG, which was compressed by the pseudoaneurysm. The combination of these techniques enabled successful treatment of the patient’s pseudoaneurysm and SVG stenosis, minimizing the risk of reoperation.

Intraoperative imaging is crucial for guiding percutaneous interventions, and TEE is the gold standard for real-time imaging during these procedures [8]. However, TEE has inherent limitations, especially when pseudoaneurysms are located near mechanical valves, as the resulting acoustic artifacts can obscure critical anatomical details [9,14]. This challenge is further compounded in patients with multiple prior cardiac surgeries, in whom an altered anatomy makes the evaluation of structures, such as pseudoaneurysms, difficult. In this case, TEE was initially used but failed to provide a clear view of the pseudoaneurysm because of interference from the mechanical aortic valve. Although TEE identified shunt flow into the pseudoaneurysm, the surrounding structures were not adequately visualized, complicating accurate device placement.

In contrast, ICE proved essential for ensuring precise device deployment, avoiding complications associated with TEE artifacts, and effectively visualizing the rim of the pseudoaneurysm. This imaging enabled accurate placement of the AVP II. By obtaining cross-sectional images from within the right atrium or near the superior vena cava, ICE provides a clear view of the ascending aorta and the pseudoaneurysm. As in the present case, the short-axis view can be instrumental; however, it should be noted that the orientation of the ICE short-axis image is reversed relative to the TEE aortic valve short-axis view, with the right atrium in the near field and the left atrium in the far field [14].

The use of ICE in interventional cardiology demonstrates its versatility and efficacy in various procedures. ICE has been used for atrial septal defect closure, catheter ablation, mitral valvuloplasty, transcatheter aortic valve replacement, and left atrial appendage closure [15]. Its ability to provide real-time high-resolution imaging within the heart makes it a valuable alternative to TEE, particularly in patients with a complex surgical history.

The successful closure of the ascending aortic pseudoaneurysm and resolution of SVG compression in this patient underscores the feasibility and safety of ICE-guided percutaneous interventions. As demonstrated in our case, ICE enabled precise device placement and real-time assessment of procedural success. This approach reduces the risk of complications and improves patient outcomes, particularly in high-risk populations in which traditional surgical options carry substantial risks.

## Conclusions

This case demonstrates the successful use of percutaneous catheter techniques to treat an ascending aortic pseudoaneurysm in a patient with a complex history of cardiac surgery. Intraoperative ICE imaging played a critical role in guiding the procedure. Percutaneous intervention should be considered a viable alternative to surgical repair in high-risk patients, offering a safe and effective treatment option. Given the imaging challenges in such cases, broader adoption of ICE should be considered in selected high-risk patients to facilitate precise device placement and reduce procedural risks.
